# Psychometric Properties of the Impact of Events Scale-Revised (IES-R) Among General Iranian Population During the COVID-19 Pandemic

**DOI:** 10.3389/fpsyt.2021.692498

**Published:** 2021-08-02

**Authors:** Hamid Sharif Nia, Harpaljit Kaur, Fatemeh Khoshnavay Fomani, Pardis Rahmatpour, Omolhoda Kaveh, Saeed Pahlevan Sharif, A. Vijayalakshmi Venugopal, Lida Hosseini

**Affiliations:** ^1^Psychiatry and Behavioral Sciences Research Center, Addiction Institute, Mazandaran University of Medical Sciences, Sari, Iran; ^2^Faculty of Business and Law, Taylor's University, Subang Jaya, Malaysia; ^3^Nursing, School of Nursing and Midwifery, Tehran University of Medical Science, Tehran, Iran; ^4^Department of Nursing, School of Nursing, Alborz University of Medical Sciences, Karaj, Iran; ^5^School of Nursing and Midwifery, Mazandaran University of Medical Science, Sari, Iran; ^6^Faculty of Business and Law, Taylor's University, Subang Jaya, Malaysia; ^7^Taylor's Law School, Taylor's University, Subang Jaya, Malaysia; ^8^School of Nursing and Midwifery, Iran University of Medical Sciences, Tehran, Iran

**Keywords:** impact of events, COVID-19 pandemic, reliability, validity, psychometric, Iran

## Abstract

**Objective:** The aim of this study was to translate and evaluate the validity and reliability of the Impact of Events Scale-Revised (IES-R) among the Iranian general population during the coronavirus disease 2019 (COVID-19) pandemic.

**Method:** This study was methodological cross-sectional. It was conducted on an Iranian public population from April to July 2020 which was during the COVID-19 pandemic. Construct validity was determined through exploratory factor analysis (EFA) and confirmatory factor analysis (CFA) with a total of 500 adults recruited *via* online data gathering. Reliability was checked through the average inter-item correlation (AIC), Cronbach's alpha, and McDonald's omega. Convergent and divergent validity was determined using Fornell and Larcker's approach.

**Results:** The results showed that the Persian version of IES-R had three factors, including intrusion (six items), avoidance (seven items), and hyperarousal (five items), that explained 59.22% of the total variance of the IES-R. The CFA findings indicated that all goodness-of-fit indices confirmed the model fit. The Cronbach's alpha, McDonald's omega, composite reliability (CR), and maximal reliability were excellent, and the three factors have good convergent validity.

**Conclusion:** The findings of this study indicated that the Persian version of the IES-R scale is efficient and useful to assess post-traumatic stress disorder among Iran general population in the COVID-19 outbreak.

## Introduction

The outbreak of COVID-19, a disease caused by the severe acute respiratory syndrome (SARS)-CoV 2 virus innovation began in late December 2019 and was named “coronavirus disease 2019” (COVID-19) by the World Health Organization. The coronavirus spread across China to other countries in <2 months (1). The countries that were affected by the outbreak of the COVID-19 disease experienced social disruption as well as dramatic loss of their people's life which presents an unprecedented challenge to public health (2, 3). Similar to the previous pandemic events, COVID-19 pandemic also caused significant psychological problems for the general population especially among susceptible individuals across the different countries (4). During the outbreak, for example, of H1N1 influenza virus in 2009 in the UK, 10–30% of the general population showed anxiety symptoms (5) whereas during SARS epidemic, 15% of the general population showed post-traumatic stress disorder (PTSD) and depression symptoms in Toronto (6). Based on literatures, the most common psychological consequences in the general population during the COVID-19 outbreak include fear of getting sick or dying, feelings of helplessness, depression, anxiety, isolation, and stigma (4, 7). Wang et al. (1) reported that during the COVID-19 outbreak, 53.8% of Chinese people suffered from moderate to severe negative psychological impact, whereby 16.5% of them had depressive symptoms, 28.8% had anxiety, and 8.1% had moderate to severe levels of stress (1). In the same vein, Mamun et al. (4) showed that 33% of the general population of Bangladesh had depression symptom and the rate of suicide ideation was 5% during the COVID-19 outbreak (4). Moreover, the findings of a systematic review study have concluded that the COVID-19 outbreak has a negative psychological impact such as anxiety (6.33–50.9%), depression (14.6–48.3%), PTSD (7–53.8%), psychological distress (34.43–38%), and stress (8.1–81.9%) on the general population (8). Furthermore, the findings of a meta-analysis study have revealed significant difference between the global depression rate in 2017 (3.44%) and 2020 (25%) (9) that may reflect the variety of stressors including long quarantine, fear of infection, frustration, boredom, insufficient resources (food, water, clothing, accommodation), insufficient information, financial losses, and stigma experienced by individuals (10). Similar to the previous pandemic events, COVID-19 leads to financial distress due to the slowdown of economic activities and the measures of health systems to control the pandemic (11, 12).

Due to the lack of vaccines or special treatment to control the COVID-19 pandemic and the rapid transmission of this disease through respiratory droplets, the only safe ways to control the disease that the World Health Organization had suggested before finding the effective vaccine were isolation, quarantine, social distance, and harnessing the community (13). Although the country policies can buffer the consequences of quarantines *via* the way they address the needs of the affected people (14), the psychological, social, and financial burden of the quarantine is identified by the systematic review studies (14–16).

Iran, one of the early epicenters, was rapidly affected by COVID-19 and emerged the surpassing infection rate in comparison with the other countries at that time (17, 18). The Irani Government officially confirmed the attack of COVID-19 in Iran on February 18, 2020 by identifying the first case of COVID-19 positive in Qom, and in a short period of time, all 31 provinces were also infected with the virus. Based on the latest statistics from WHO, 2,215,445 people were infected and 66,327 died as per April 18, 2021 (17, 19, 20).

Living in a vastly different context than the other countries who were affected by the COVID-19 outbreak, Iranian people experienced a more challenging pandemic (21, 22), as the outbreak has affected the Iranian people more than any other country. This is due to the ongoing decade-long US-led sanctions (23), and though the Iran government has taken effort to launch the social and mental support programs, the ability of the government to protect the nation financially during the quarantine was not sufficient (22). Iran has experienced at least four waves of the COVID-19 infection due to different reasons such as the poor decisions regarding to relax lockdown, the availability of personal protective equipment (PPE), cultures, labor and employment conditions, the ease of working from home and maintaining a living in a pandemic, and the information in both mainstream and social media (21, 23). However, only a few studies have explored the impact of COVID-19 on the general Iranian population. Vahedian-Azimi et al. (24) assessed the rate of stress, anxiety, and depression among four groups of Iranian society including community population, patients with COVID-19, medical staff, and medical students in the initial stage of the COVID-19 outbreak, and they reported that the medical students and patients with COVID-19 had higher stress, anxiety, and depression than the medical staff and community population. In the same vein, Shahriarirad et al. (25) evaluated the burden of psychological problems on the Iranian general population during the outbreak of COVID-19, and they found that 15.1 and 20.2% of the population had clinically significant depressive and anxiety symptoms (25). Since the focus of the media and the health system generally in the world is on the ramification of epidemic, the psychological effect of COVID-19 are largely ignored. Therefore, given the psychological burden of this disease in the world, it is necessary that its effects be properly investigated using accurate and appropriate scale.

The Impact of Event Scale-Revised (IES-R) designed by Weiss et al. is a short and self-reporting questionnaire with three clusters of symptoms including hyperarousal, intrusion, and avoidance subscales specifically tailored to investigate post-traumatic stress reactions and particularly PTSD in the aftermath of a trauma (26). The IES-R is translated and validated in different language and context including Indonesian (27), Swedish (28), French (29), Chinese (30), Japanese (31), Greek (32), Dutch (33), South Korean (34), and Sri Lankan (35). Based on the review of studies, it has not been translated into Persian. Identifying the impacts of the COVID-19 event among the general population is beneficial to improve the public health (36, 37), and better management of the future crisis and outbreak (38). Therefore, considering the importance of identifying the impacts of the COVID-19 event in the general population and lack of validated measure to investigate it, this study was designed to translate and evaluate the validity and reliability of the IES-R in a general population during the COVID-19 pandemic.

## Methods

### Design

This methodological cross-sectional study was conducted on the public population from April to July 2020 during the COVID-19 pandemic to evaluate the validity and reliability of the Persian version of the IES-R.

### Measurement

In this study, we used two questionnaires which included the demographic questionnaire and Persian version of IES-R. The demographic questionnaire composed of personal information (age, gender, marital status, educational level, and employment status) and two questions about personal and family history of having COVID-19.

The original version of IES-R is a self-report, short, and easily administered questionnaire to assess PTSD based on the criteria of the Diagnostic and Statistical Manual of Mental Disorders, Fourth Edition (DSM-IV) and can be used with both healthy and frail individuals who are exposed to any specific traumatic event. This scale consists of 22 items with three factors including “intrusion” (difficulty in staying asleep, dissociative experiencing, similar to flashbacks) with eight items, “avoidance” (the tendency to avoid thoughts or reminders about the incident) with eight items, and “hyperarousal” (irritated feeling, angry, difficulty in sleep onset) with six items. In addition to the three subscale scores, the IES-R total with the sum of the three subscale scores is also obtained (39). The IES-R is scored on a 5-point Likert-type scale from 0 (not at all) to 4 (extremely) (26) which means that the total score range calculated is between 0 and 88 and the cut-off of 33 indicates a high risk of PTSD symptomatology.

### Translation

Regarding the validity of translation process, the IES-R was translated based on the standards recommended in the guidelines (40). Firstly, we obtained the written permission from the co-author of the developer of the scale, Professor Charles Marmar *via* email. Secondly, two English-Persian translators translated the IES-R independently. A panel of experts, which included two professional translators, evaluated the two translation versions and created one Persian translation of IES-R. A back-translation was then independently completed by the two Persian to English translators who were blinded to the English version of the IES-R. The panel of experts then compiled and compared the results of the back-translation with the original instrument to detect the differences and similarities between the original instrument and the back-translated version. A pilot test was conducted with 30 participants from the general population who were recruited online using convenience sampling. The Persian version of the questionnaire was completed online by the participants, who were asked to identify the ambiguous items and suggest preferred statements if needed. The pilot testing resulted in no item changing.

### Construct Validity

The construct validity of the Persian version of IES-R scale was evaluated by exploratory and confirmatory factor analysis. Based on the recommendation of MacCallum et al., the sample size should be at least 200 cases for factor analysis (41). A total of 500 adult people were recruited *via* the online data gathering, out of which 250 samples were used for exploratory factor analysis (EFA) and another 250 samples were used to evaluate confirmatory factor analysis (CFA). The inclusion criteria for participants were adults (age >18) who were willing to participate in this study. Online data gathering was performed for this section where the online questionnaire was created *via* Google Forms and its URL link was sent by email or social networking applications such as Telegram channel or WhatsApp groups. Data were then extracted into an Excel file from the Google Forms.

Construct validity was evaluated using Maximum Likelihood Exploratory Factor analysis (MLEFA) with Promax rotation. The Kaiser–Meyer–Olkin test (KMO) and the Bartlett's test of sphericity were used to check the appropriateness of the study sample and the model. The number of factors was determined based on parallel analysis and Exploratory Graph Analysis (EGA). Items with absolute loading values of at least 0.3 were considered appropriate (42).

The presence of an item in a latent factor was determined based on a factor loading of almost 0.3, which was estimated using the following formula: CV = 5.152 ÷ √ (n – 2), where CV was the number of extractable factors and “n” was the sample size. The number of latent factors was estimated using Horn's parallel analysis. Next, items with communalities <0.2 were excluded from the EFA. For assessment of the structural factors, a CFA was conducted using the maximum-likelihood method and the most common goodness of fit indices. The model fitness was assessed according to the Root Mean Square of Error of Approximation (RMSEA), Comparative Fit Index (CFI), Parsimonious Comparative Fit Index (PCFI), Parsimonious Normed Fit Index (PNFI), Incremental Fit Index (IFI) and CMIN/DF. In CFA, items with a standardized factor loading lower than 0.5 were removed from the model.

#### Convergent and Divergent Validity

The convergent and divergent validity of the IES-R were estimated using Fornell and Larcker's approach (43). The Average Variance Extracted (AVE) and Maximum Shared Squared Variance (MSV) were estimated to assess the convergent and discriminant validity of the extracted factors. To establish convergent validity, (a) AVE should be >0.5, and (b) Composite Reliability (CR) should be >0.7. To meet the discriminant validity criteria, the MSV of each construct should be less than its AVE (44).

#### Reliability

Reliability of the scale was evaluated using internal consistency and construct reliability (CR). Internal consistency was assessed using the average inter-item correlation (AIC), Cronbach's alpha and McDonald's omega (45). If the Cronbach's alpha was higher than 0.7, it indicates that the scale has internal consistency (46). CR was calculated using the structural equation model analysis as an alternative to Cronbach's alpha coefficient and is acceptable if it was >0.7 (47).

### Multivariate Normality and Outliers

It is necessary to mention that skewness (±3) and kurtosis (±7) were used to determine items such as the normal distribution, outliers, missing data, and the univariate and multivariate distributions of data individually. In this process, the Mahalanobis d-squared (*p* < 0.001) was used for evaluating the existence of multivariate outliers and Mardia coefficient of multivariate kurtosis (<8) was used for evaluating the existence of multivariate normality (47). Also, multiple imputations were used to evaluate the missing data, after which, the data was replaced by the average participant response (48).

SPSS_26_, SPSS-R menu_2_, AMOS_24_, and JASP_0.13.1.0_ software were used for performing all of the statistical calculations.

### Ethical Approval

The protocol of this study was approved by the Mazandaran University of Medical Sciences Research Ethics Committee (IR.MAZUMS.REC.1399.7461). The study aims, number of items, time of completing the scale, the researcher's affiliation and email for queries, ethical code of study inserted in the first page of online questionnaire, and informed participants that their participations was voluntary and that their responses would be published anonymously as group data.

## Results

The mean and standard deviation for the age of the 500 adults were 34.61 ± 9.2 years. Other demographic characteristics of the participants are provided in Table 1.

**Table 1:** Demographic characteristics of participants (*n* = 500).

**Variables**	***N* (%)**
**Gender**	
Female	342 (68.4)
Male	158 (31.6)
**Marital status**	
Single	222 (44.4)
Married	278 (55.6)
**Education level**	
Under diploma	19 (3.8)
Diploma	57 (11.4)
Bachelor	227 (45.4)
Master/PhD	197 (39.4)
**Employment**	
Unemployed	122 (24.4)
Employed	291 (58.2)
Student	87 (17.4)
**History of COVID-19**	
Yes	127 (25.4)
No	373 (74.6)
**Family history of COVID-19**	
Yes	223 (44.6)
No	277 (55.4)

**Figure 1:**
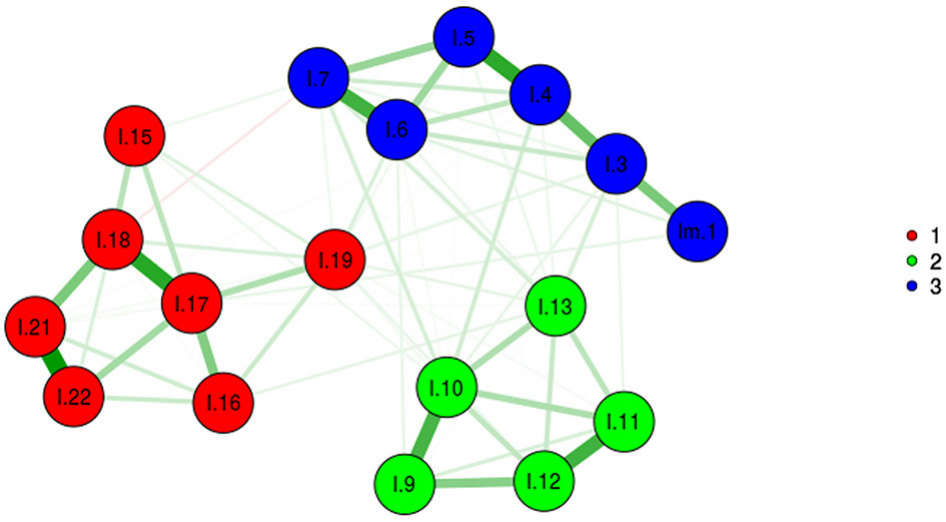
Exploratory graph analysis.

**Figure 2:**
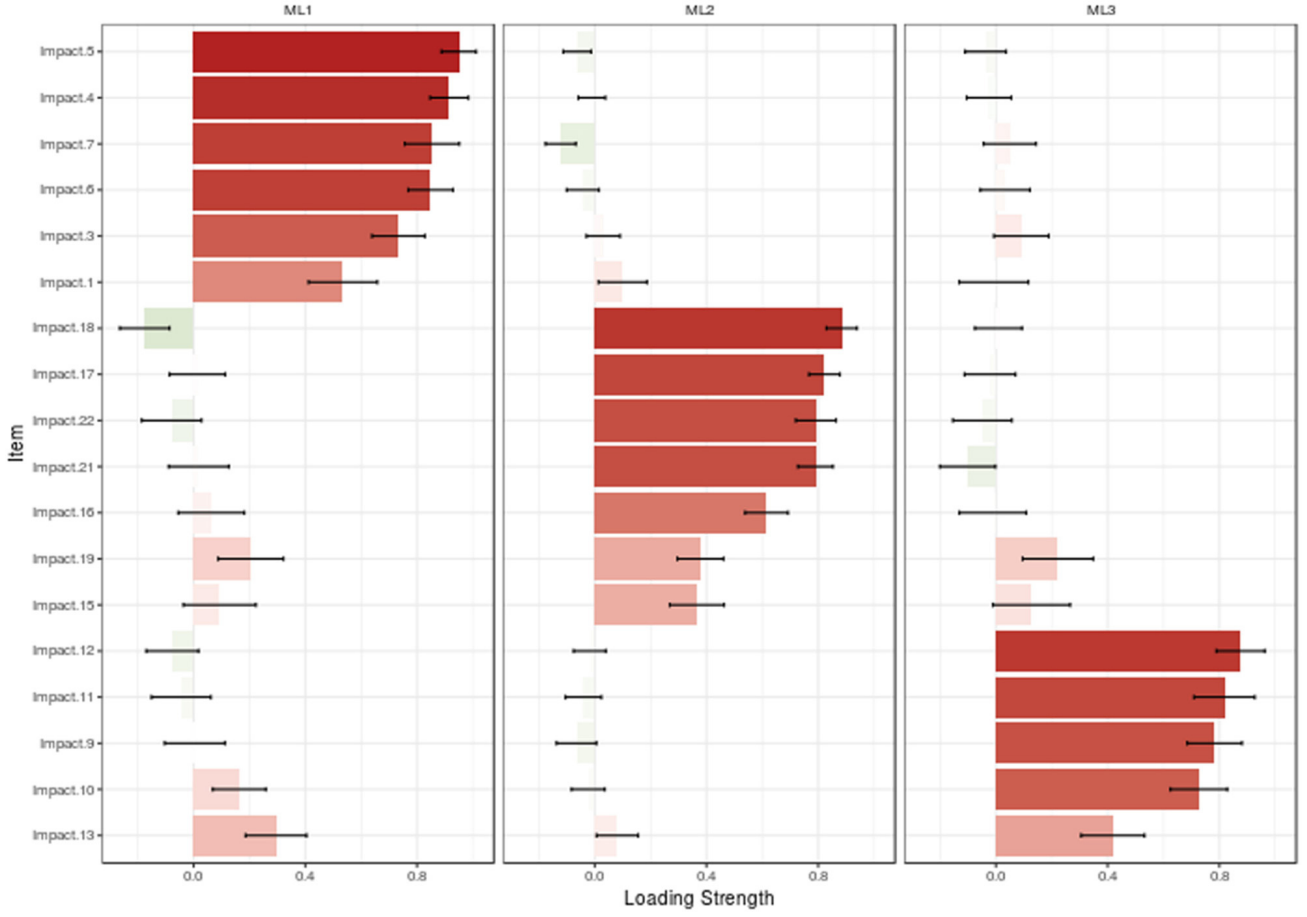
Loading strength of items in factors.

In MLEFA, the KMO test value was 0.931 and Bartlett's test value was 6,022.415 (*p* < 0.001). Given the three extracted factor approach (Figures 1, 2), MLEFA revealed a three-factor structure for IES-R. The Eigenvalues and percent of variances of these three factors are shown in Table 2. These three factors explained 59.22% of the total variance of the IES-R in this sample.

**Table 2:** Exploratory factors extracted of IES-R (*n* = 250).

**Factors**	**Q_**n**_. Item**	**Factor loading**	^*^h^**2**^	**Eigenvalue**	**%Variance**
Intrusion	5. Pictures about it popped into my mind.	0.949	0.799	4.41	23.11
	4. I thought about it when I didn't mean to.	0.915	0.797		
	7. I had waves of strong feelings about it.	0.853	0.699		
	6. I found myself acting or feeling like I was back at that time.	0.848	0.725		
	3. Other things kept making me think about it	0.733	0.668		
	1. Any reminder brought back feelings about it.	0.534	0.342		
Avoidance	18. I tried not to think about it.	0.884	0.659	3.38	19.21
	17. I stayed away from reminders of it	0.822	0.674		
	22. I tried not to talk about it.	0.791	0.551		
	21. I tried to remove it from my memory.	0.790	0.584		
	16. I felt as if it hadn't happened or wasn't real.	0.615	0.412		
	19. I was aware that I still had a lot of feelings about it, but I didn't deal with them.	0.378	0.448		
	15. I avoided letting myself get upset when I thought about it or was reminded of it.	0.365	0.246		
Hyper-arousal	12. I had trouble concentrating.	0.876	0.655	3.19	16.90
	11. I had trouble falling asleep.	0.818	0.581		
	9. I felt irritable and angry.	0.783	0.576		
	10. I was jumpy and easily startled.	0.726	0.703		
	13. Reminders of it caused me to have physical reactions, such as sweating, trouble breathing, nausea, or a pounding heart	0.418	0.494		

* *h^2^: Communalities*.

**Figure 3:**
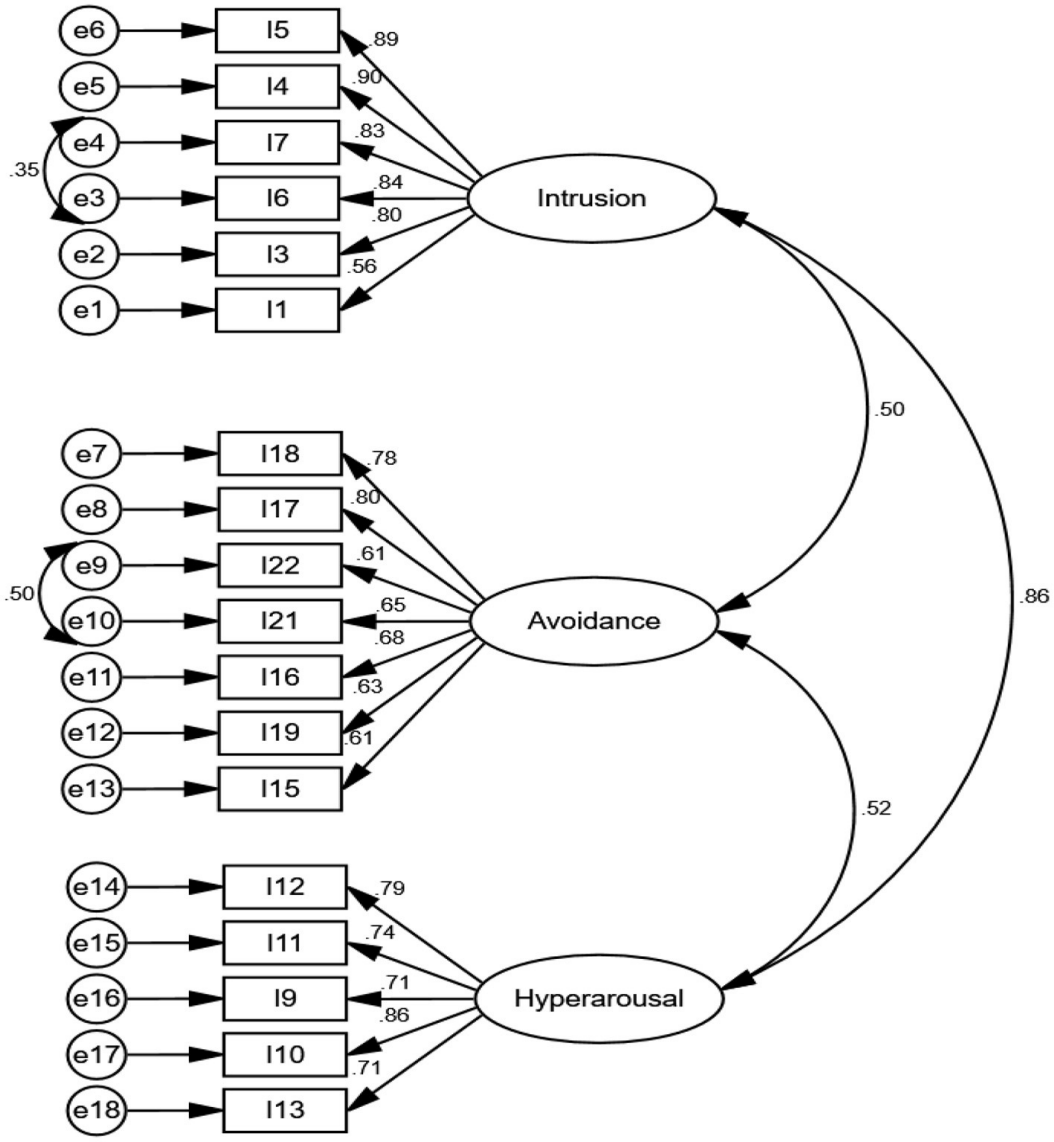
First—order CFA of IES-R (*n* = 250).

**Figure 4:**
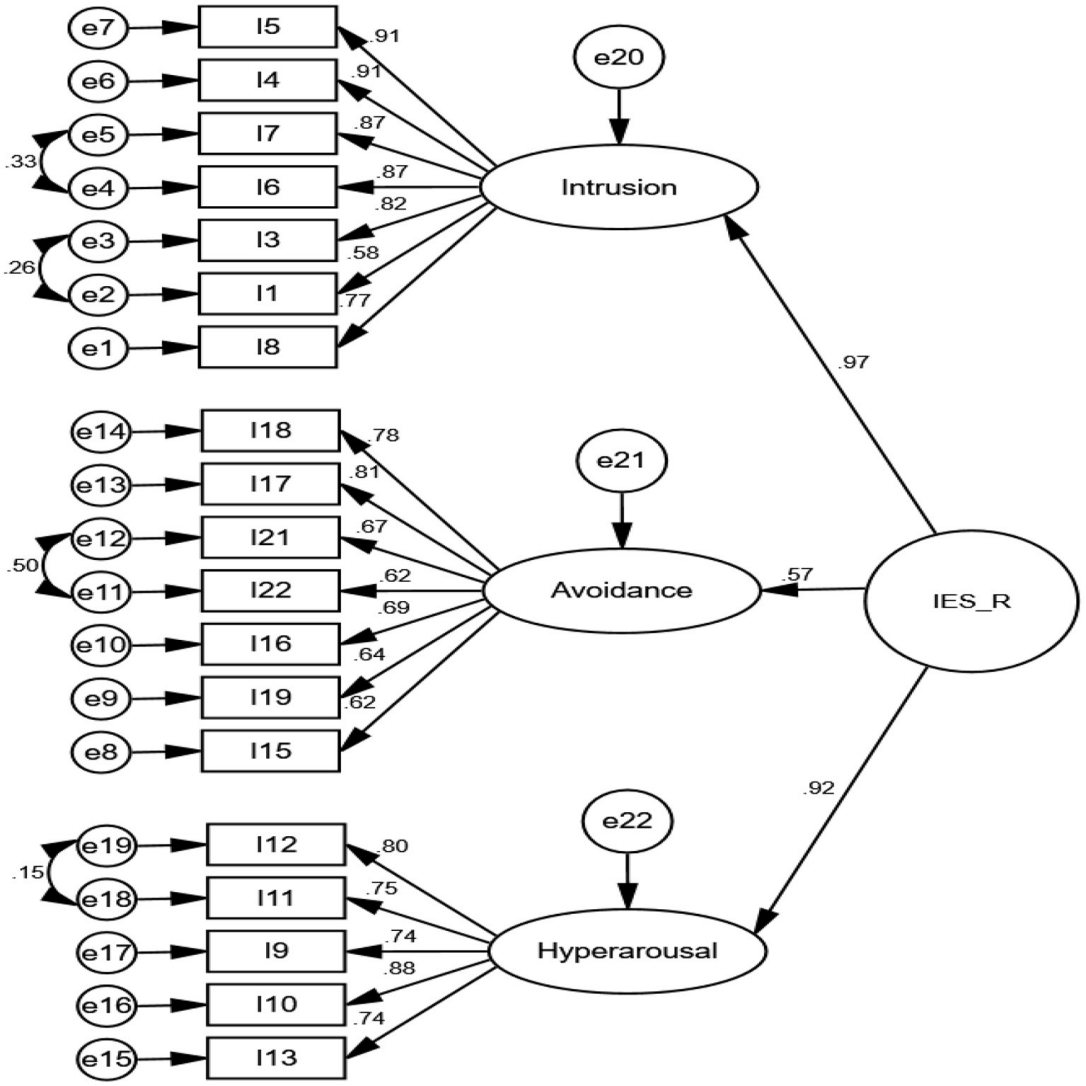
Second—order CFA of IES-R (*n* = 250).

The findings of CFA indicate that all goodness-of-fit indices confirmed the model fit as provided in Table 3. The Cronbach's alpha, McDonald's omega, CR, and maximal reliability of three extracted factors of the IES-R were excellent. The AIC values of factors were good. Regarding convergent and discriminant validity, the AVE of two factors was more than the MSV and shows that the factors have good convergent but no discriminant validity (Table 4). The covariances between factors were more than 0.50, indicating a latent variable behind them (see Figure 3). Thus, a second-order CFA was performed (see Figure 4).

**Table 3:** Fit indices of the first and second order confirmatory factor analysis of the IES-R (*n* = 250).

**Indices**	**χ^2^**	**df**	***P*-value**	**CMIN/DF**	**RMSEA (CI 90%)**	**PNFI**	**PCFI**	**TLI**	**IFI**	**CFI**
First order CFA	276.689	130	<0.001	2.12	0.067 (0.056–0.078)	0.771	0.806	0.939	0.949	0.948
Second order CFA	285.414	130	<0.001	2.19	0.069 (0.58–0.080)	0.768	0.803	0.935	0.945	0.945

*DF, Degree of freedom; PCFI, Parsimonious Comparative Fit Index; PNFI, Parsimonious Normed Fit Index; CMIN/DF, Minimum Discrepancy Function divided by Degrees of Freedom; RMSEA, Root Mean Square Error of Approximation; TLI, Tuker-Lewis Index; and CFI, Comparative Fit Index, IFI, Incremental Fit IndexFitness indexes, PNFI, PCFI (>0.5); TLI, IFI, CFI (>0.9), RMSEA (<0.08), CMIN/DF (<3 good, <5 acceptable)*.

**Table 4:** The indices of the convergent, discriminant validity, and internal consistency of IES-R for the first-order CFA (*n* = 250).

	**CR**	**AVE**	**MSV**	**MaxR(H)**	**Alpha**	**Omega**	**AIC**
Intrusion	0.919	0.658	0.733	0.937	0.92 (0.91–0.93)	0.74	0.62
Avoidance	0.858	0.466	0.275	0.871	0.86 (0.84–0.87)	0.71	0.46
Hyper arousal	0.875	0.584	0.733	0.886	0.87 (0.85–0.89)	0.82	0.58

## Discussion

In the present study, we aimed to introduce the Persian version of IES-R as a screening of self-reported questionnaire that gauge subjective distress for various specific life events in an Iranian public population with the current COVID-19 pandemic and to verify its validity, reliability, and factor structure with different psychometric properties. In general, the results of the current study support the applicability, reliability, and validity of the Persian version of IES-R in the Iranian population sample. The results of the EFA explained 59.22% of the total variance of the IES-R in this sample, compared with 49% in a sample of Malaysian women (49).

Based on the findings, the Persian version of the IES-R showed a clear factor structure with three factors, namely, intrusion (six items), avoidance (seven items), and hyperarousal (five items). The Cronbach's alpha coefficients for the three subscales of the IES-R were high (ranging from 0.84 to 0.93) that were in line with those seen in other past studies (49–51), indicating good internal consistency. In addition, other evaluation of internal consistency tested by McDonald's omega, CR, and maximal reliability, all demonstrated that the Persian IES-R had excellent satisfactory reliability. Moreover, the AIC values of the factors were good, ranging from 0.46 to 0.62.

In this study, the first factor is “intrusion” with six items which addressed mind preoccupation—unwanted thinking about it, a wave of strong feeling, feeling back in that time, thinking about it and returning feeling due to other things, and dreaming about it. Horowitz et al. (52) indicated that intrusion is a preoccupation with a traumatic experience, frequent thoughts and images about the experience, related feelings about the experience, troubled dreams about the experience, and a frequent need to talk about the experience. The COVID-19 pandemic has created continuous traumatic events such as fear of contagion, risk of death, vaccination, and uncertainty of the various measures adopted to counteract the spread of infection, over a long period of time worldwide, significantly impacting mental health and generating increased risk of stress-related disorders and PTSD symptoms (32, 39, 51, 53–55).

The second factor is “avoidance” with seven items that referred to trying not to think, staying away from reminders, trying to remove from memory, trying not to talk about it, feeling like it had not happened or was not real, and not allowing yourself to feel upset when thinking about it. Sundin and Horowitz (56) stated that avoidance is a mechanism for dealing with tragic life events that works by denying the meaning and consequences of the event and behavioral inhibitions (56).

The third factor is “hyperarousal” with five items relating to trouble concentrating, trouble falling asleep, feeling irritable and angry, feeling jumpy and being easily startled, and physical reactions such as sweating, trouble breathing, nausea, or a pounding heart. A change in arousal is one of the four dimensions of PTSD, and the negative impact of the PTSD symptomatology in daily life extending to social interaction, work activity, and family life is observed frequently (39). This is further supported by more recent studies by Sami and Hallaq (57) and Peng et al. (58) who found that both avoidance and hyperarousal were risk factors for depressive symptoms (57, 58).

In line with this study, Craparo et al. (51) in the Italian samples that were involved in the natural disaster (flood victims) showed a clear factor structure with three independent dimensions: intrusion (four items), avoidance (four items), and hyperarousal (seven items) with 15 items (51), while in this study, the number of items was identified as 18 items (51). Another study reported IES-6 three-factor solution in a sample of victims of bank robbery (50). Norhayati and Aniza (49) demonstrated three constructs with 10 items of the Malay version of IES-R among postpartum women. According to the present study, the three-factor structure for the IES-R was clearly shown, with the slightly different items loaded in each subscale from the original IES-R, which is due to cultural differences and differences in the nature of the COVID-19 pandemic.

It is noteworthy that currently, Vanaken et al. (59) validated the Impact of the Event Scale with modifications for COVID-19 (IES-COVID19). Their target population was 380 university students during the early stage of the COVID-19 outbreak. CFA showed the factor structure of the IES-COVID19 as being similar to the original IES with two latent factors—intrusion and avoidance. Based on the limitations of their study and the impact of this disease on the general population, they recommended to validate this scale in the general population (59). The findings of this study indicated that the Persian version of the IES-R scale is efficient and useful to assess PTSD among the general population in the COVID-19 outbreak. This self-reported questionnaire manages well the assessment of PTSD during the period when the Iran government gradually eased the lockdown restriction with intensification of the social distancing policy in early April, encouraging people to stay at home and abide with strict health protocols (60). Thus, the data collected in this study will be able to emphasize the results associated with the perception of intrusion, avoidance and hyper arousal from respondents who experience this pandemic for the first time, to perceive it as highly traumatic, especially in quarantine. These quarantine measures kept many people in isolation, restricting their movements and interaction which caused significant physiological effects and may impact the mental well-being of the individuals (37, 61), influencing individual's emotional state (62). Though the end impact of COVID-19 is unclear at this point in time, its psychological effects such as distress about getting infected, infection to their family members and loved ones, fear of death, anger, sleep issues, and anxiety, all of which affect the emotional and physical health of individuals, suggesting high risk of PTSD development (62, 63). Based on recent studies, healthcare providers, psychologists, and psychiatrists are also at high risk of exposure to COVID-19, not only are they faced with the increased level of psychological distress, in addition to longer work time, more work responsibility, but they are exposed to increasing numbers of patients and constant updates of hospital procedures (64–67). Thus, many of the healthcare providers developed burnout, PTSD, anxiety, and depression after the cessation of the pandemic, and to avoid such problems in the future, the Persian version IES-R could be used as a tool for early intervention to measure the subjective psychological distress triggered by any traumatic event.

## Limitations

Although the sample assessed in the present study was large, convenience sampling may be limited in its ability to reach all groups of the population, especially the elderly population and individuals with no Internet or without access to social media such as WhatsApp, Telegram, or email, which is one of the most important limitations of this study. Since the elderly are more affected by the COVID-19 pandemic due to their vulnerability and it was difficult to access them through social networks, it is recommended that this scale be considered in this group. In addition, since, the underlying mental health issues might definably have had an effect, this study was limited by the absence of the diagnostic interviews for the assessment of PTSD symptoms in the validation of the Persian version of the IES-R. Due to the unprecedented pandemic, data was collected online, and this data collection is a limitation for itself. In addition, another limitation of this current study was the absence of any possible form of support by qualified professionals for participants of the study who were positively screened for PTSD.

## Conclusion

The current study may be of interest as it aims at validating one of the most widely used self-report measures to assess PTSD symptomatology in the Persian language, in a large sample of adults during the COVID-19 pandemic in Iran. The scale had an acceptable construct validity and reliability. It has three factors with 18 items that explained 59.22% of the total variance of the IES-R in adults during the COVID-19 pandemic. This scale could be beneficial for researchers, psychologists, and healthcare providers to assess PTSD symptom, as the IES-R is aimed at assessing and investigating the traumatic burden of the COVID-19 pandemic.

## Data Availability Statement

The data that support the findings of this study are available from the corresponding author upon reasonable request.

## Ethics Statement

The studies involving human participants were reviewed and approved by Mazandaran University of Medical Sciences Research Ethics Committee (IR.MAZUMS.REC.1399.7461). The patients/participants provided their written informed consent to participate in this study.

## Author Contributions

All authors listed have made a substantial contribution in design the study, data collection, analyzing the results, writing the manuscripts, and approving the final manuscript.

## Conflict of Interest

The authors declare that the research was conducted in the absence of any commercial or financial relationships that could be construed as a potential conflict of interest.

## Publisher's Note

All claims expressed in this article are solely those of the authors and do not necessarily represent those of their affiliated organizations, or those of the publisher, the editors and the reviewers. Any product that may be evaluated in this article, or claim that may be made by its manufacturer, is not guaranteed or endorsed by the publisher.
